# 1979. Waning of humoral immunity depending on the types of COVID-19 vaccine

**DOI:** 10.1093/ofid/ofac492.1604

**Published:** 2022-12-15

**Authors:** So Yun Lim, Ji Yeon Kim, Jiwon Jung, Sung-Cheol Yun, Sung-Han Kim

**Affiliations:** Asan medical center, Seoul, Seoul-t'ukpyolsi, Republic of Korea; Asan medical center, Seoul, Seoul-t'ukpyolsi, Republic of Korea; Asan Medical Center, Seoul, Seoul-t'ukpyolsi, Republic of Korea; Asan medical center, Seoul, Seoul-t'ukpyolsi, Republic of Korea; Asan medical center, Seoul, Seoul-t'ukpyolsi, Republic of Korea

## Abstract

**Background:**

There are limited data on the rates of the waning of antibody levels after two-dose and booster vaccination according to the different platforms of COVID-19 vaccines.

**Methods:**

We enrolled healthcare workers (HCWs) in a tertiary care hospital who received homologous two-dose vaccination, followed by a homologous or heterologous booster mRNA vaccine. SARS-CoV-2 S1-specific IgG was measured using ELISA. A linear mixed regression model was used to compare the slope from the peak antibody titer to the lowest antibody titers 3 months after vaccination.

**Results:**

A total of 113 HCWs (BNT162b2 (n=48 [42%]), ChAdOx1 nCoV-19 (n=52 [46%]) or mRNA-1273 (n=13 [12%])) were enrolled in this prospective cohort study. More gradual antibody waning was observed over 3 months with the two-dose ChAdOx1 nCoV-19 (ChAdOx1) than with the two-dose BNT162b2 or mRNA-1273 (p< 0.001 and p=0.001, respectively). In addition, homologous mRNA-1273 booster induced a more durable antibody response than homologous BNT162b2 booster (p< 0.001) or heterologous ChAdOx1-BNT162b2 booster (p< 0.001).

**Figure:**
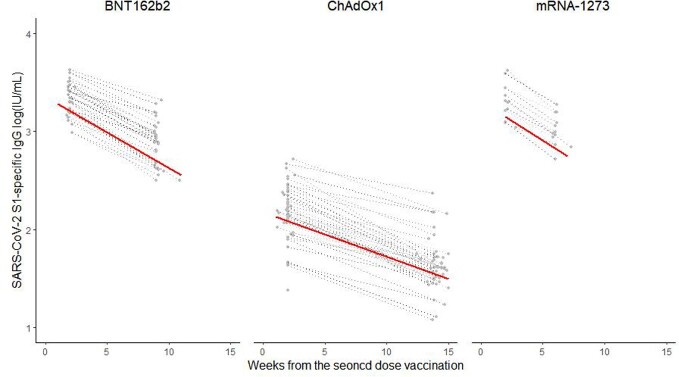

**Figure:**
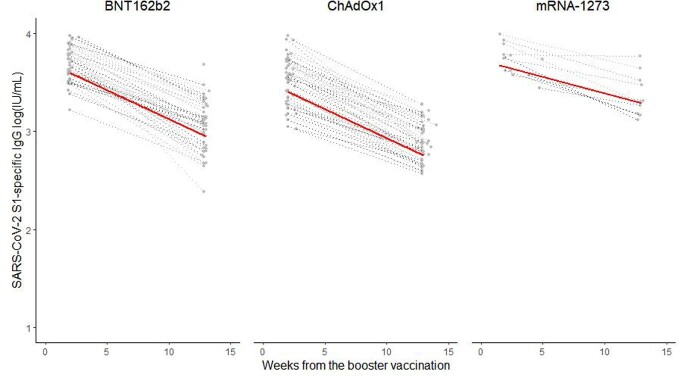

**Figure:**
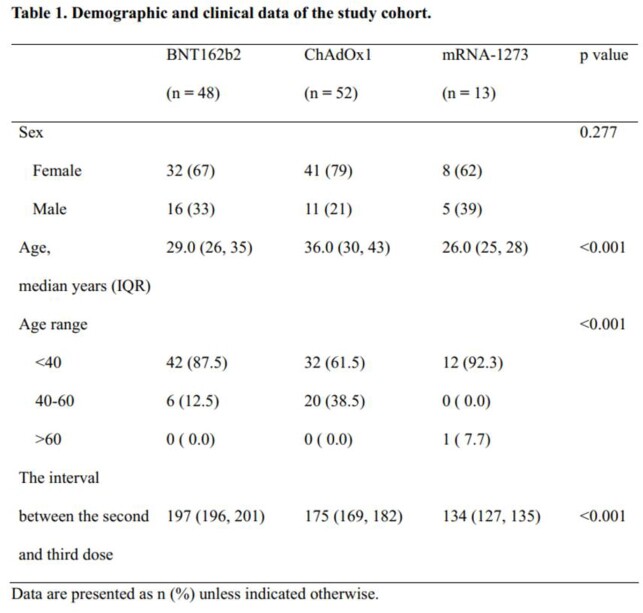

**Conclusion:**

2-dose homologous ChAdOx1 vaccination or homologous mRNA-1273 booster appears to induce more-durable antibody responses than 2-dose homologous mRNA vaccination, homologous BNT162b2 booster, or 2-dose ChAdOx1 followed by BNT162b2 booster.

**Disclosures:**

**All Authors**: No reported disclosures.

